# Genome-wide gene expression profiling suggests distinct radiation susceptibilities in sporadic and post-Chernobyl papillary thyroid cancers

**DOI:** 10.1038/sj.bjc.6603938

**Published:** 2007-08-21

**Authors:** V Detours, L Delys, F Libert, D Weiss Solís, T Bogdanova, J E Dumont, B Franc, G Thomas, C Maenhaut

**Affiliations:** 1 Institute of Interdisciplinary Research, School of Medicine, Univertisté Libre de Bruxelles (ULB), Campus Erasme, CP602, route de Lennik 808, Brussels B-1070, Belgium; 2 Institute of Endocrinology and Metabolism, Kiev 04114, Ukraine; 3 Service d'Anatomie et de Cytologie Pathologiques, APHP (Hôpital Ambroise Paré), Faculté de Médecine Paris Ile de France Ouest, Université Versailles Saint-Quentin en Yvelines, 9 Avenue Charles de Gaulle, Boulogne 92100, France; 4 South West Wales Cancer Institute/Swansea Clinical School, Singleton Hospital, Sketty Lane, Swansea SA2 8QA, UK

**Keywords:** thyroid cancers, Chernobyl, radiation susceptibility, microarray

## Abstract

Papillary thyroid cancers (PTCs) incidence dramatically increased in the vicinity of Chernobyl. The cancer-initiating role of radiation elsewhere is debated. Therefore, we searched for a signature distinguishing radio-induced from sporadic cancers. Using microarrays, we compared the expression profiles of PTCs from the Chernobyl Tissue Bank (CTB, *n*=12) and from French patients with no history of exposure to ionising radiations (*n*=14). We also compared the transcriptional responses of human lymphocytes to the presumed aetiological agents initiating these tumours, *γ*-radiation and H_2_O_2_. On a global scale, the transcriptomes of CTB and French tumours are indistinguishable, and the transcriptional responses to *γ*-radiation and H_2_O_2_ are similar. On a finer scale, a 118 genes signature discriminated the *γ*-radiation and H_2_O_2_ responses. This signature could be used to classify the tumours as CTB or French with an error of 15–27%. Similar results were obtained with an independent signature of 13 genes involved in homologous recombination. Although sporadic and radio-induced PTCs represent the same disease, they are distinguishable with molecular signatures reflecting specific responses to *γ*-radiation and H_2_O_2_. These signatures in PTCs could reflect the susceptibility profiles of the patients, suggesting the feasibility of a radiation susceptibility test.

An increased incidence of thyroid carcinomas in children was first noticed in Belarus and Ukraine 4 years after the 1986 Chernobyl accident (Baverstock *et al*, 1992; Kazakov *et al*, 1992). Increased incidence has been observed since then in people exposed to fallout during childhood in these regions (United Nations Scientific Committee of the Effect of Atomic Radiation, 2000; Mahoney *et al*, 2004). The aggressiveness and morphology of these tumours (over 95% classified on the basis of their pathology as papillary thyroid carcinomas (PTCs)) appear to be related to the age of the patients at the time of the accident and to the lag between the accident and diagnosis, that is, the latency of the cancers (Williams *et al*, 2004).

Radiation is the only proven cause of PTC so far. Although the cause of PTC in patients not exposed to radiation remains uncertain, a growing body of evidence suggests that H_2_O_2_ could play a role in the absence of radiation. Indeed, it is a potent DNA-damaging agent produced in large amounts during thyroid hormone synthesis (Corvilain *et al*, 2000). It causes DNA damage (guanine oxidation, single- and double-strand breaks) in human lymphocytes (Turner *et al*, 2003), hamster ovarian cells (Dahm-Daphi *et al*, 2000; Mondello *et al*, 2002), and in human, dog and sheep thyroid cells in primary culture (Chico Galdo *et al*, 2006). Hydrogen peroxide is believed to destroy follicular thyroid cells in myxoedematous endemic cretinism (Kohrle *et al*, 2005) and to cause cancers in the thyroid of Tg-*α*_1B_AR mice (Ledent *et al*, 1997). Lack of protective systems, peroxiredoxin or glutathione peroxidases, in knockout mice lead to cancer (Neumann *et al*, 2003; Lee *et al*, 2006). Transfection of an H_2_O_2_-generating system transform epithelial cells (Chu *et al*, 1996). The spontaneous somatic mutation rate in normal mice and rat thyroid cells is substantially higher than in liver and lung cells (Corvilain *et al*, 1994). With a turnover of 8.5 years in adults (Coclet *et al*, 1989), thyrocytes have time to accumulate H_2_O_2_-induced DNA damages. Hydrogen peroxide has been found to play a role in several human cancers (Quinn *et al*, 2006). Thus, a number of arguments support a role of H_2_O_2_ in the initiation of PTC, and in particular in patients not exposed to radiation.

The vast majority of PTCs harbour either a BRAF mutation (45%; Xing, 2005) or a RET/PTC rearrangement (35% in adults; Nikiforov, 2002), which are generally mutually exclusive (Soares *et al*, 2003). Both gene alterations result in the constitutive activation of the RAS–RAF–MAPK signalling pathway (Kimura *et al*, 2003; Soares *et al*, 2003). Gene-expression signatures separating BRAF from RET/PTC tumours have been reported, but the number of genes involved varies from a few dozens (Frattini *et al*, 2004) to several thousands (Giordano *et al*, 2005). Although early reports pointed at a lower BRAF mutation frequency in Chernobyl patients, recent evidence suggests that the BRAF mutation is associated with age and is more prevalent among older Chernobyl patients and/or among patients with longer latency tumours (Kumagai *et al*, 2004; Lima *et al*, 2004; Powell *et al*, 2005; Rosenbaum *et al*, 2005). Several research teams have reported higher frequencies of RET/PTC rearrangements in post-Chernobyl patients (Nikiforov *et al*, 1997). These higher frequencies could result from the fact that radiation induces double-strand breaks, and thus rearrangements rather than point mutations (Dahm-Daphi *et al*, 2000), or possibly to a differing molecular profile in childhood *vs* adult papillary carcinomas (Powell *et al*, 2005). The induction of RET/PTC rearrangements after *in vitro* irradiation of immortalised thyroid cells (Caudill *et al*, 2005) supports the former explanation. Whether the two best-characterised genetic alterations found in PTC are involved in a radiation signature remains an open question. In addition, radiation induces other unknown alterations.

In this paper, we have compared the gene-expression profiles of PTCs from adult French patients with no history of exposure to radiation and from adult Ukrainian patients exposed to Chernobyl fallout during childhood, and asked whether there is a gene-expression signature distinguishing radiation-induced from sporadic cancers. Our preliminary investigation suggested the absence of a large-scale radiation signature (Detours *et al*, 2005). We extend it here by using a more recent microarray technology, by covering more genes, by studying more patients and by establishing results with a wider range of statistical methods. We confirm that French and Chernobyl Tissue Bank (CTB) tumours have the same overall expression profiles and have indistinguishable BRAF and RET/PTC frequencies.

We also compared the transcriptional responses of human cells to the two most likely aetiological agents of CTB and French tumours; *γ*-radiation and H_2_O_2_. The similarity of CTB and French tumours is mirrored by the similarity of the transcriptional responses to *γ*-radiation and H_2_O_2_. However, subtle expression differences are exploitable to accurately classify these tumours according to their origin. Part of these expression differences includes genes involved in the differential response to H_2_O_2_ and radiation, and genes involved in homologous recombination which suggests that different—and detectable—susceptibility profiles lead to sporadic and radiation—induced PTC.

## MATERIALS AND METHODS

### Transcriptional and genetic data

Paired samples of tumoral and adjacent non-tumoral thyroid tissues were obtained from the CTB (www.chernobyltissuebank.com) and from patients undergoing surgery for thyroid disease at the Ambroise Pare Hospital (Boulogne, France). French tissues were immediately frozen in liquid nitrogen and stored at −80°C until use. Diagnoses were made by the Department of Pathology at the Ambroise Pare Hospital or by the International Pathology Panel of the CTB. The protocol received approval from the Ethics Committees of the institutions. The detail of BRAF-RET/PTC status determination, RNA processing and microarray data preprocessing is available in Supplementary information file S3. Microarray data are available from the Gene Expression Omnibus (www.ncbi.nlm.nih.gov/geo), accession number GSE3950.

### Comparison of microarray platforms

Jarzab *et al* (2005) data were downloaded from www.genomika.pl/thyroidcancer/PTCCancerRes.html. We used the original MAS 5.0 normalised expression levels, took the log_2_ of expression ratios and averaged over patients. The probes of the two platforms could be matched on the basis of their Entrez IDs for 4203 genes.

### Unsupervised classification

Hierarchical clustering was computed with the R language function hclust with Ward linkage. Multidimensional scaling was computed with the R function isoMDS. Both methods were fed Pearson correlation distances as input.

### Supervised classification

Support vector machine classification was run with linear kernel and cost=1 using the rfe 0.2 and e1071 1.5.9 packages for R. The generalised partial least-square (GPLS) implementation from package gpls 1.1.0 (Ding and Gentleman, 2004) for R was run with default parameters. Prediction analysis of microarray (Tibshirani *et al*, 2002) was run with threshold values in {1.0, 1.1, 1.2, …, 3.0} using pamr 1.25 for R. The random forest classification used default parameters from R package randomForest 4.5.12 (Zhang *et al*, 2003). Generalised partial least-square and random forest (RF) were combined with an external genes selection procedure focusing on the *n* genes with the highest absolute *t*-statistics, with *n* in {1, 2^1^, 2^2^, …, 2^13^}. We adopted the inner/outer cross-validation scheme described in details in Ruschhaupt *et al* (2004) and implemented in the package MCRestimate 1.3.0 to prevent parameter and gene selection biases (Ambroise and McLachlan, 2002). Note that a simpler split-sample validation, in which samples are not recycled as in the current cross-validation protocol, would be suboptimal here because of the limited availability of CTB samples (Simon *et al*, 2003). A 13-fold cross-validation protocol with each round including parameter and gene selection, and classification was run. At each one of the 13 rounds, the best parameters (including signature size) were estimated by running a nested (inner) 12-fold cross-validation for each combination of parameters. Table 2 presents averages over 10 repetitions of the entire inner/outer cross-validation, each based on a different random 13-fold partitions of the data. The random error was computed by averaging the error of five runs of the complete classification procedure on data with CTB and French labels randomly assigned to samples. The same protocol was used for the classification on the basis of the 118 genes *γ*-radiation *vs* H_2_O_2_ signature, except that the number of genes, *n*, was chosen in {1, 5, 10, …, 118} and that the tested prediction analysis of microarray (PAM) thresholds were in {0.1, 0.2, …, 3.0}. Classifications on the basis of DNA repair signatures were run without gene selection, and therefore without inner cross-validation. The PAM threshold was set to 0.5. All *P*-values were derived by running 1000 times the complete cross-validation with CTB and French labels assigned randomly to samples and counting how many runs produced classification error below the error obtained on the actual data.

### Derivation of the *γ*-radiation *vs* H_2_O_2_ signature

We downloaded the Supplementary data set S2 of Amundson *et al* (2005) from the *Oncogene* web site (www.nature.com/onc/index.html). Genes with expression values differing by 1.5-fold between the 2.5 Gy *γ*-radiation- and H_2_O_2_ (200 *μ*M)-treated TK6 cells were selected. To remove immune system-related genes, we downloaded the gcrma-processed version of the GNF human gene atlas (Su *et al*, 2004; symatlas.gnf.org), which contains expression profiles of normal tissues in most organs. We performed an unpaired two class Significance Analysis of Microarrays (SAM; Tusher *et al*, 2001) with class no. 1 including immune system-related tissues and white blood cells and class no. 2 including all other tissues. We selected the 20% top-ranking genes, which were all significant at *q*<0.05, and removed them from the *γ*-radiation and H_2_O_2_ signature.

## RESULTS

### Expression profiles and gene alteration status of PTCs from France and from the Chernobyl Tissue Bank

Expression profiles were determined for the tumours of 14 patients from France with no documented history of exposure to radiation, and 12 tumours from the Chernobyl Tissue Bank (see online Materials and Methods). CTB tumours are papillary cancers collected in young people who were exposed to the Chernobyl accident ((Thomas *et al*, 2000), see patient information, Table 1). There are 9 tumours of classical subtype, 4 of follicular subtype and 1 of trabecular subtype among the 14 French PTC samples. There are 8 classical, 3 follicular and 1 solid subtypes among the 12 CTB PTC samples. Three French and four CTB mRNA samples (PTC6, PTC7, PTC11 and S405, S420, S422, S423) were reused from our earlier study (Detours *et al*, 2005).

**Figure 1:**
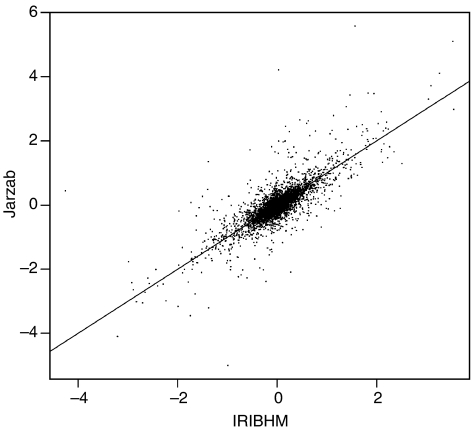
IRIBHM *vs* Jarzab *et al*. (2005) microarray data. Pearson correlation between patient-averaged log_2_ tumour/normal ratios of the two studies is 0.72.

The mRNA expression profiles of all tumours were determined with 12 000 EST (8000 genes) cDNA microarrays using patient-matched nontumoural adjacent tissues as controls. To assess the quality of the data, we compared our expression ratios averaged over samples with those of Jarzab *et al* (2005), who used the Affymetrix® platform. Pearson's correlation measured on the ∼4000 genes available and expressed in both platforms was 0.72 (Figure 1).

Tumours were screened for the presence of a RET/PTC rearrangement and for BRAF V600E mutation (Table 1). A RET/PTC rearrangement was found in 42% (5/12) of the CTB tumours and in 21% (3/14) of the French tumours. The difference between the two groups is not significant according to Fisher's exact test. The BRAF mutation is found in comparable proportions in French (36%, 5/14) and CTB tumours (41%, 5/12). None of these alterations was detected in 30% (8/26) of the tumours.

### Chernobyl Tissue Bank and French PTCs have similar overall expression profiles

**Figure 2:**
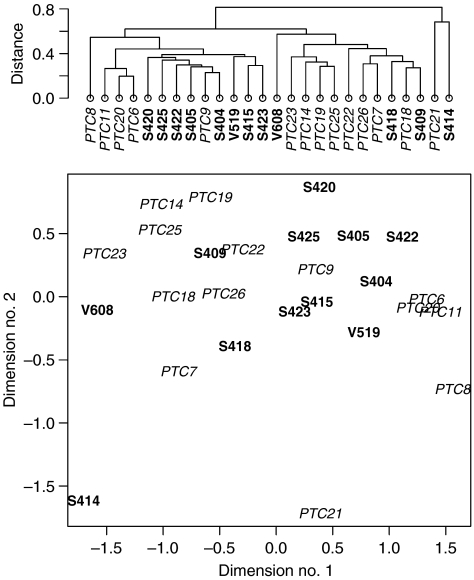
Global expression profiles. Top panel: hierarchical clustering on the basis of all genes. Bottom panel: multidimensional scaling on the basis of all genes. Distances in the two dimensions space were on average distorted by 11% compared to the actual 8000 dimensions gene space distances. Chernobyl Tissue Bank tumours are in bold font, French tumours in italics.

We first searched for global expression differences between CTB and French PTCs, that is, extensive differences detectable when all the genes present on our arrays are considered. Hierarchical clustering based on all genes did not reveal a clear separation between these two classes of PTCs (Figure 2, upper panel).

Multidimensional scaling collapses the high-dimensional genes space into two dimensions while preserving the distance relationships between all pairs of samples (Figure 2, lower panel). Figure 2 confirms that French and CTB tumours have similar expression profiles when compared on a global scale although CTB tumours form a more compact group.

### Four supervised classification algorithms find multigenes signatures separating CTB from French PTCs

The absence of separation between CTB and French PTCs on the basis of all genes or at the level of individual genes, does not exclude that these tumours are distinguishable on the basis of a subset of genes. We investigated this possibility with a supervised classification approach (details in Materials and Methods). To strengthen the reliability of our conclusions, all the results were reproduced with four linear classification procedures: linear kernel support vector machines (LKSVM), GPLS, PAMs and RF. Each one included or was combined with a gene selection procedure, that is, a procedure to uncover multigenes signatures including as few genes as possible. All four approaches were tested using a rigorous inner/outer cross-validation procedure (Materials and Methods). It guaranteed that classification testing was performed on independent samples not used for classifier training. The cross-validation results are presented in Table 2A.

**Figure 3:**
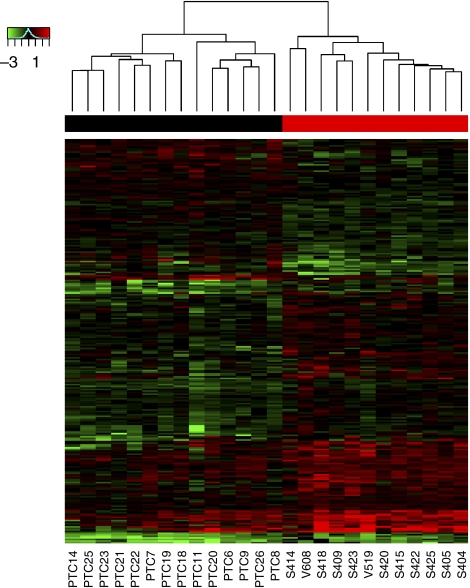
Top 256 most classifying genes according to GPLS/*t*-statistics. Chernobyl Tissue Bank samples are in red and French samples in black in the top colour bar. Data are ordered with two-way hierarchical clustering for the sake of display clarity.

The best performer was GPLS. It misclassified 17% of CTB tumours as French PTC, 7% of French PTCs as CTB, resulting in an overall error rate of 12%. Running the same classification on data in which the ‘CTB’ and ‘French’ labels were randomly assigned to the 26 tumour samples led to high error rates of 45% (*n*=5, s.d.=12%, see Materials and Methods), as expected for random classification of slightly unbalanced classes (12 CTB and 14 French samples). Thus, the low error rates were unlikely to result from artefacts, including data overfitting. Figure 3 shows the 256 most classifying genes found by GPLS/*t*-statistics trained on all 26 samples (corresponding genes listed in Supplementary Table S1). The optimal signature size varied among the different cross-validation runs from one gene to several thousands of genes, with a median of 256 genes. Such limited stability is widespread, including in large studies (Ein-Dor *et al*, 2005; Michiels *et al*, 2005). The three other classification procedures, LKSVM/RFE, RF/*t*-test and PAM produced a global error of 15, 23 and 27%, respectively. Thus, undirected selections of classifying genes lead to separation CTB and French tumours.

Note that if the classification results were confined to a subtype, the accuracy would not be as low as 15%, it would be greater than 35%—the classical subtype is the largest, representing 65% of our tumours.

### Hydrogen peroxide and *γ*-radiation elicit similar transcriptional responses in lymphocytes

Because hydrogen peroxide, H_2_O_2_, is produced at high levels during thyroid hormone synthesis (Corvilain *et al*, 2000) and is a well-known DNA-damaging agent, we investigated the possibility that in the absence of an obvious external risk factor, for example radiation, French cancers must have occurred as a result of H_2_O_2_ exposure.

**Figure 4:**
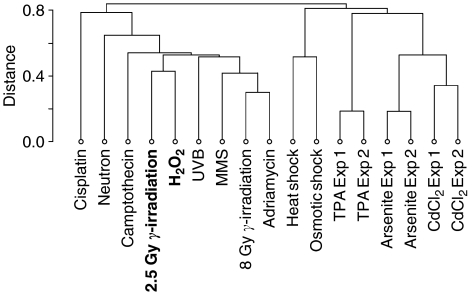
Hierarchical clustering of transcriptional responses of the B-lymphocyte TK6 cell line to various stress agents. Expression data are from Amundson *et al* (2005). The responses to 200 *μ*M of H_2_O_2_ and 2.5 Gy *γ*-radiation cluster together. Abbreviations: MMS, methyl methanesulphonate; TPA, 12-*O*-tetradecanoylphorbol 13-acetate; UVB, ultraviolet (280−320 nm). The suffixes ‘Exp1’ and ‘Exp2’ stand for replicated experiments.

Amundson *et al* (2005) measured with microarrays the transcriptional responses of a B-lymphocyte cell line, TK6, to 13 stress agents. These included 10 DNA-damaging agents: H_2_O_2_, radiation (neutron and *γ-*rays at 2.5 and 8 Gy), adriamycin, arsenite, campothecin, CdCl_2_, cisplatin, methyl methanesulphonate and UVB (280−320 nm). We downloaded the expression data published with the paper and produced the hierarchical clustering shown in Figure 4 (see online Materials and Methods). The responses to 200 *μ*M H_2_O_2_ and to 2.5 Gy *γ-*radiation clustered together, that is, among 12 stress agents, including 10 DNA-damaging agents, *γ*-radiation at 2.5 Gy elicited the transcriptional response that was the closest to that of H_2_O_2_. We concluded that these similar transcriptional responses reflect similar damages in the cells.

### Chernobyl Tissue Bank and French tumours are accurately classified on the basis of genes regulated differently in *γ*-radiation and H_2_O_2_ responses

The transcriptional responses to *γ*-radiation and H_2_O_2_ are broadly similar; however, some genes are expressed differently between the two *in vitro* assays. We reasoned that these expression differences may mirror subtle underlying *γ*-radiation and H_2_O_2_ susceptibility differences between CTB and French tumours that could be used for classification.

We found 293 genes in the 1451 published by Amundson *et al* (2005) with a fold change greater than 1.5 between the *γ*-radiation (2.5 Gy) and the H_2_O_2_ responses (200 *μ*M). These responses were measured in B lymphocytes, whereas our goal was to classify thyroid tumours. Thus, we removed immune system-specific genes from the set of 293 genes (see online Materials and Methods). This filtering left 162 genes. Among them, 118 were spotted on our microarrays. They are listed in Supplementary Table S2 and will be referred to thereafter as the *γ*-radiation *vs* H_2_O_2_ signature. Note that it was derived independently of our PTC data.

Next, we applied the same four classification algorithms as above except that only the independently selected 118 genes were used. Error rates (Table 2B) were comparable to those obtained in Table 2A, where the classifying genes were selected from a list of 8000. Again, all four algorithms classified the tumours with an error rate ⩽27%, GPLS/*t*-test and LKSVM/RFE being the most accurate with an error rate of 15%. This result shows a relation between the *γ*-radiation *vs* H_2_O_2_ signature and CTB and sporadic carcinomas distinction, which could reflect the underlying aetiology of CTB and French tumours.

### Chernobyl Tissue Bank and French tumours are accurately classified on the basis of 13 genes involved in homologous recombination

To focus better on which elements of the DNA-damage response may differ between CTB and French tumours, we investigated if genes involved in the different DNA repair mechanisms led to accurate classification. We collected from the Human DNA Repair Genes database (Wood *et al*, 2001, 2005), all the genes known to be involved in base-excision repair, mismatch-excision repair, nucleotide-excision repair, homologous recombination and nonhomologous end joining. The signature from each one of these five repair mechanisms was then used to classify the CTB and French tumours. These signatures contain few genes and were compiled from a source curated by DNA repair experts. Therefore, we skipped the gene selection step, which in turn alleviates the need for time-consuming internal cross-validation. The resulting computational gain made it tractable to run an additional statistical control: all five classification tasks were rerun 1000 times with the CTB and French labels randomly assigned to the tumours to estimate *P*-values, that is, the odds that the classification error was as low as the one observed with the actual data. Besides this, the classification proceeded exactly as above.

The classification error rates were high for base-excision repair, mismatch-excision repair and nonhomologous end joining, regardless of the algorithm (not shown). The nucleotide-excision repair signature produced an error rate of 27% with RF, but ∼50% with GPLS, PAM and LKSVM. In contrast, the homologous recombination signature (Table 3) led to a classification below 31% for all four procedures, below 20% for two and equal to 15% for LKSVM (Table 2C).

The *P*-value for RF, 0.064, was slightly above the 0.05 significance standard. All the other *P*-values were highly significant and remained below 0.02 after adjusting for the fact that five classification tasks were being examined (using Bonferonni correction, i.e., multiplying the *P*-values by 5). This suggests that homologous recombination, which repairs double-strand breaks, operates differently in CTB and French tumours or in the associated normal tissues. None of the homologous recombination signature genes are part of the 118 genes of the *γ*-radiation *vs* H_2_O_2_ signature. Thus, the homologous recombination and *γ*-radiation *vs* H_2_O_2_ signatures are nonoverlapping. They are thus two different signatures supporting a link between radiation and the CTB/French PTC expression differences.

## DISCUSSION

We compared French and CTB tumours at the level of their global expression profiles, that is, of their overall phenotype. Hierarchical clustering and multidimensional scaling failed to uncover a large-scale difference between them. Note that, would such difference exist, our preliminary study (Detours *et al*, 2005) would have revealed it. Thus, the conclusion of pathologists that sporadic and radiation-induced PTCs are the same type of lesions is supported by expression data.

The similarity of expression profiles on a global scale, as observed with hierarchical clustering performed on all genes, does not preclude that small groups of genes differ between these profiles. Supervised classification is the tool of choice to evaluate whether a group of genes can be exploited to discriminate different classes of tumours (Allison *et al*, 2006). Four linear classification algorithms assigned the tumours to the French or CTB groups with an error ranging from 12 to 27%, and ⩽15% for two algorithms. These figures are typical of properly designed microarray studies (Ntzani and Ioannidis, 2003), and compare very favourably with histopathological diagnosis accuracy in the field of thyroid tumours (Baloch *et al*, 2001; Hegedus, 2004; Clary *et al*, 2005). The stability of the gene lists uncovered through supervised classification is problematic, even in studies using hundreds of samples (Ein-Dor *et al*, 2005; Michiels *et al*, 2005). Clearly, much larger studies will be needed to list exactly and exhaustively the discriminating genes, and validate them over a larger group. Nevertheless, our results strongly suggest that such genes exist: accurate classification of CTB and French tumours is possible on the basis of their expression profiles.

Initial reports of a low BRAF mutation frequencies in post-Chernobyl tumours (Nikiforova *et al*, 2004) and of a large impact of BRAF on gene expression (Giordano *et al*, 2005) raised the possibility of a radiation damage signature based on the mutational status of the tumours. The frequency of BRAF V600E mutation was similar, 38%, in our French and CTB tumours. Our analysis does not exclude the possibility of other damage signatures yet to be identified.

Radiation is a proven causing factor for PTC and a number of arguments support the view that H_2_O_2_-induced damage also contributes to initiate these tumours (see Introduction). Taking advantage of published data on the transcriptional responses of human lymphocytes to 13 stress agents (Amundson *et al*, 2005), we investigated how similar the responses to H_2_O_2_ and *γ*-radiation are. We found that among 10 genotoxic agents, H_2_O_2_ at 200 *μ*M elicits the response most similar to that of radiation at 2.5 Gy. This strengthens the argument for H_2_O_2_ as a PTC-causing agent, as this similarity most probably mirrors a similarity of the damage inflicted by H_2_O_2_ and radiation. This similarity is in line with the finding that French and CTB tumours have similar global profiles. Interestingly, Xiong *et al* (2005) demonstrated that the number of chromatid breaks per cell following *γ*-irradiation was significantly higher in the lymphocytes of 57 PTC patients with no documented exposure to radiation than in the lymphocytes of healthy controls. This difference could be related to impaired homologous recombination as the 18067T allele variant of XRCC3 was more frequent in 134 thyroid cancer patients than in 166 healthy patients in another study (Sturgis *et al*, 2005).

Transcriptional responses to H_2_O_2_ and *γ*-radiation are similar relatively to other responses to genotoxic agents. However, 118 genes regulated differently in response to H_2_O_2_ and radiation were uncovered and could be used to classify CTB and French tumours with an error as low as 15%. This is straightforward evidence that at least some of the genes associated with these tumours are also associated with the response to their presumed respective aetiological agent.

Next, we investigated whether French and CTB tumours could be classified on the basis of five signatures covering the genes involved in the five major DNA repair mechanisms: base-excision repair, mismatch-excision repair, nucleotide-excision repair, homologous recombination and nonhomologous end joining (Wood *et al*, 2001, 2005). The homologous recombination signature, which shares no genes with the H_2_O_2_ *vs γ*-radiation signature, led to classification errors ranging from 15 to 31%. None of the other four signatures led to accurate classification. The specificity for the homologous recombination effect, and the good classification of CTB and French tumours using the 118 genes regulated differently in response to H_2_O_2_ and radiation, make unlikely the confounding effect of age- or ethnicity-related factors. The fact that homologous recombination is involved in double-strand break repair fits the notion that radiation causes more double-strand breaks than H_2_O_2_. Nevertheless, although potential confounders are controlled for by the use of patient-matched adjacent tissues, they are not formally ruled out in our study. This will become possible in the future as tumours from younger Ukrainian patients born after 1987 become available.

Thus, several independent gene-expression signatures separate our CTB and sporadic PTCs. These subtle expression differences between CTB and French tumours must be interpreted in light of the fact that the tumours investigated were removed >15 years after the Chernobyl accident. Thus, any discriminating gene-expression signature had to be sustained over this time interval. DNA damage resulting from radiation, however, is typically mostly repaired within a time scale of hours. Consequently, either the reported signatures are ‘damage signatures’, that is, they are late results, from radiation-induced DNA damage (e.g. non- or incorrectly repaired damage), and/or they are ‘susceptibility signatures’, that is, they mirror radiation susceptibility factors pre-existing to the accident. The fact that one of the signature relies on the relative response to the two postulated causing agents (*γ*-rays and H_2_O_2_), and that the other relies on double-strand break repair genes, suggests that these signatures are related to the tumour-initiating mechanisms. This and the longlasting presence of these signatures support the susceptibility signature model. The recent finding that different TP53 alleles are associated with radiation exposure in adult PTC from Russian-Ukrainian patients (Rogounovitch *et al*, 2006) also supports this view. The susceptibility model, and the corollary that radiation susceptibility varies among individuals, may partly explain why only a minority of the population most exposed to radiation in Ukraine and Belarus developed PTC.

Thus, we interpret our findings as evidence for different and detectable cancer susceptibility factors underlying CTB and French tumours, which leads to several testable predictions. Expression ratios of tumours with respect to patient-matched adjacent tissues were measured. Hence, we could uncover susceptibility signatures only to the extent that they manifest themselves differently in the cancers and their adjacent tissues. We anticipate that the direct comparison of expression levels instead of expression ratios could lead to a stronger signature, possibly involving more genes. In addition, a radiation susceptibility signature could be present in healthy cells of any type in post-Chernobyl cancer patients. This, then, suggests the possibility of developing an expression-based *in vitro* test for radiation susceptibility. Finally, large-scale studies could uncover the genetic or epigenetic variations underlying the phenotypic differences reported in this paper. These concepts and approaches may apply to other types of cancers.

## Figures and Tables

**Table 1:** Patient information and gene alterations

**Sample ID**	**Origin**	**Sex**	**Age in 1986**	**Age at operation**	**BRAF**	**RET/PTC**
PTC11	FR	F	22	37	−	−
PTC14	FR	M	17	32	−	−
PTC18	FR	F	NA	59	+	−
PTC19	FR	M	54	68	−	+
PTC20	FR	F	54	68	+	−
PTC21	FR	F	39	54	−	+
PTC22	FR	F	44	60	−	−
PTC23	FR	M	17	33	−	−
PTC25	FR	F	49	60	−	−
PTC26	FR	F	36	47	+	−
PTC6	FR	M	24	37	−	−
PTC7	FR	F	13	29	+	−
PTC8	FR	M	22	36	−	+
PTC9	FR	F	24	38	+	−
S404	CTB	F	1	16	−	−
S405	CTB	F	1	16	−	+
S409	CTB	F	11	28	+	−
S414	CTB	F	16	33	−	+
S415	CTB	M	12	28	+	−
S418	CTB	M	10	27	+	−
S420	CTB	F	12	28	−	−
S422	CTB	M	15	31	+	−
S423	CTB	F	5	22	+	−
S425	CTB	M	3	19	−	+
V519	CTB	F	2	18	−	+
V608	CTB	F	15	32	−	+

F=female; FR=France; CTB=Chernobyl Tissue Bank; M=male; NA=not available; PTC=papillary thyroid cancer.

**Table 2:** Error rates for supervised classification

	**French error**	**CTB error**	**Global error**	
*(a) Classification based on all genes*				
GPLS	17	7	12	
PAM	25	29	27	
RF	33	14	23	
LKSVM	25	7	15	
				
*(b) Classification based on H*_*2*_*O*_*2*_ *vs γ-radiation signature*				
GPLS	8	21	15	
PAM	25	29	27	
RF	42	7	23	
LKSVM	25	7	15	
	**French error**	**CTB error**	**Global error**	***P***
*(c) Classification based on homologous radiation signature*				
GPLS	17	21	19	0.0038
PAM	25	21	23	<0.001
RF	42	21	31	0.063
LKSVM	8	21	15	0.0038

CTB=Chernobyl Tissue Bank; GPLS=generalised partial least-square; LKSVM=linear kernel support vector machines; PAM=prediction analysis of microarray; RF=random forest.

Classification and validation procedures are described in Materials and Methods.

**Table 3:** Homologous recombination gene signature

**Symbol**	**Name**
XRCC2	X-ray repair complementing defective repair in Chinese hamster cells 2
SHFM1	Split hand/foot malformation (ectrodactyly) type 1
RAD51C	RAD51 homologue C (*Saccharomyces cerevisiae*)
MUS81	MUS81 endonuclease
RAD51L1	RAD51-like 1 (*S. cerevisiae*)
RAD51	RAD51 homologue (RecA homologue, *Escherichia coli*) (*S. cerevisiae*)
RAD50	RAD50 homologue (*S. cerevisiae*)
RAD54B	RAD54B homologue
RAD54L	RAD54-like (*S. cerevisiae*)
NBS1	Nijmegen breakage syndrome 1 (nibrin)
RAD52	RAD52 homologue (*S. cerevisiae*)
XRCC3	X-ray repair complementing defective repair in Chinese hamster cells 3
BRCA1	Breast cancer 1, early onset

Only homologous recombination genes represented on our microarrays are listed (see main text).

## References

[bib1] Allison DB, Cui X, Page GP, Sabripour M (2006) Microarray data analysis: from disarray to consolidation and consensus. Nat Rev Genet 7: 55–65 16369572 10.1038/nrg1749

[bib2] Ambroise C, McLachlan GJ (2002) Selection bias in gene extraction on the basis of microarray gene-expression data. Proc Natl Acad Sci USA 99: 6562–6566 11983868 10.1073/pnas.102102699 PMC124442

[bib3] Amundson SA, Do KT, Vinikoor L, Koch-Paiz CA, Bittner ML, Trent JM, Meltzer P, Fornace Jr AJ (2005) Stress-specific signatures: expression profiling of p53 wild-type and -null human cells. Oncogene 24: 4572–4579 15824734 10.1038/sj.onc.1208653

[bib4] Baloch ZW, Hendreen S, Gupta PK, LiVolsi VA, Mandel SJ, Weber R, Fraker D (2001) Interinstitutional review of thyroid fine-needle aspirations: impact on clinical management of thyroid nodules. Diagn Cytopathol 25: 231–234 11599106 10.1002/dc.2044

[bib5] Baverstock K, Egloff B, Pinchera A, Ruchti C, Williams D (1992) Thyroid cancer after Chernobyl. Nature 359: 21–22 10.1038/359021b0 1522880

[bib6] Caudill CM, Zhu Z, Ciampi R, Stringer JR, Nikiforov YE (2005) Dose-dependent generation of RET/PTC in human thyroid cells after *in vitro* exposure to gamma-radiation: a model of carcinogenic chromosomal rearrangement induced by ionizing radiation. J Clin Endocrinol Metab 90: 2364–2369 15671095 10.1210/jc.2004-1811

[bib7] Chico Galdo V, Massart C, Jin L, Vanvooren V, Caillet-Fauquet P, Andry G, Lothaire P, Dequanter D, Friedman M, Van Sande J (2006) Acrylamide, an *in vivo* thyroid carcinogenic agent, induces DNA damage in rat thyroid cell lines and primary cultures. Mol Cell Endocrinol 257–258: 6–14 10.1016/j.mce.2006.06.003 16859826

[bib8] Chu R, Lin Y, Reddy KC, Pan J, Rao MS, Reddy JK, Yeldandi AV (1996) Transformation of epithelial cells stably transfected with H_2_O_2_-generating peroxisomal urate oxidase. Cancer Res 56: 4846–4852 8895731

[bib9] Clary KM, Condel JL, Liu Y, Johnson DR, Grzybicki DM, Raab SS (2005) Interobserver variability in the fine needle aspiration biopsy diagnosis of follicular lesions of the thyroid gland. Acta Cytol 49: 378–382 16124165 10.1159/000326169

[bib10] Coclet J, Foureau F, Ketelbant P, Galand P, Dumont JE (1989) Cell population kinetics in dog and human adult thyroid. Clin Endocrinol (Oxf) 31: 655–665 2627756 10.1111/j.1365-2265.1989.tb01290.x

[bib11] Corvilain B, Collyn L, Van Sande J, Dumont JE (2000) Stimulation by iodide of H(2)O(2) generation in thyroid slices from several species. Am J Physiol Endocrinol Metab 278: E692–E699 10751204 10.1152/ajpendo.2000.278.4.E692

[bib12] Corvilain B, Laurent E, Lecomte M, Van Sande J, Dumont JE (1994) Role of the cyclic adenosine 3′,5′-monophosphate and the phosphatidylinositol-Ca^2+^ cascades in mediating the effects of thyrotropin and iodide on hormone synthesis and secretion in human thyroid slices. J Clin Endocrinol Metab 79: 152–159 8027219 10.1210/jcem.79.1.8027219

[bib13] Dahm-Daphi J, Sass C, Alberti W (2000) Comparison of biological effects of DNA damage induced by ionizing radiation and hydrogen peroxide in CHO cells. Int J Radiat Biol 76: 67–75 10665959 10.1080/095530000139023

[bib14] Detours V, Wattel S, Venet D, Hutsebaut N, Bogdanova T, Tronko MD, Dumont JE, Franc B, Thomas G, Maenhaut C (2005) Absence of a specific radiation signature in post-Chernobyl thyroid cancers. Br J Cancer 92: 1545–1552 15812549 10.1038/sj.bjc.6602521 PMC2362019

[bib15] Ding B, Gentleman RC (2004) Classification using generalized partial least squares. J comput Graphical Stat 14: 280–298

[bib16] Ein-Dor L, Kela I, Getz G, Givol D, Domany E (2005) Outcome signature genes in breast cancer: is there a unique set? Bioinformatics 21: 171–178 15308542 10.1093/bioinformatics/bth469

[bib17] Frattini M, Ferrario C, Bressan P, Balestra D, De Cecco L, Mondellini P, Bongarzone I, Collini P, Gariboldi M, Pilotti S, Pierotti MA, Greco A (2004) Alternative mutations of BRAF, RET and NTRK1 are associated with similar but distinct gene expression patterns in papillary thyroid cancer. Oncogene 23: 7436–7440 15273715 10.1038/sj.onc.1207980

[bib18] Giordano TJ, Kuick R, Thomas DG, Misek DE, Vinco M, Sanders D, Zhu Z, Ciampi R, Roh M, Shedden K, Gauger P, Doherty G, Thompson NW, Hanash S, Koenig RJ, Nikiforov YE (2005) Molecular classification of papillary thyroid carcinoma: distinct BRAF, RAS, and RET/PTC mutation-specific gene expression profiles discovered by DNA microarray analysis. Oncogene 24: 6646–6656 16007166 10.1038/sj.onc.1208822

[bib19] Hegedus L (2004) Clinical practice. The thyroid nodule. N Engl J Med 351: 1764–1771 15496625 10.1056/NEJMcp031436

[bib20] Jarzab B, Wiench M, Fujarewicz K, Simek K, Jarzab M, Oczko-Wojciechowska M, Wloch J, Czarniecka A, Chmielik E, Lange D, Pawlaczek A, Szpak S, Gubala E, Swierniak A (2005) Gene expression profile of papillary thyroid cancer: sources of variability and diagnostic implications. Cancer Res 65: 1587–1597 15735049 10.1158/0008-5472.CAN-04-3078

[bib21] Kazakov VS, Demidchik EP, Astakhova LN (1992) Thyroid cancer after Chernobyl. Nature 359: 21 10.1038/359021a0 1522879

[bib22] Kimura ET, Nikiforova MN, Zhu Z, Knauf JA, Nikiforov YE, Fagin JA (2003) High prevalence of BRAF mutations in thyroid cancer: genetic evidence for constitutive activation of the RET/PTC–RAS–BRAF signaling pathway in papillary thyroid carcinoma. Cancer Res 63: 1454–1457 12670889

[bib23] Kohrle J, Jakob F, Contempre B, Dumont JE (2005) Selenium, the thyroid, and the endocrine system. Endocr Rev 26: 944–984 16174820 10.1210/er.2001-0034

[bib24] Kumagai A, Namba H, Saenko VA, Ashizawa K, Ohtsuru A, Ito M, Ishikawa N, Sugino K, Ito K, Jeremiah S, Thomas GA, Bogdanova TI, Tronko MD, Nagayasu T, Shibata Y, Yamashita S (2004) Low frequency of BRAFT1796A mutations in childhood thyroid carcinomas. J Clin Endocrinol Metab 89: 4280–4284 15356022 10.1210/jc.2004-0172

[bib25] Ledent C, Denef JF, Cottecchia S, Lefkowitz R, Dumont J, Vassart G, Parmentier M (1997) Costimulation of adenylyl cyclase and phospholipase C by a mutant alpha 1B-adrenergic receptor transgene promotes malignant transformation of thyroid follicular cells. Endocrinology 138: 369–378 8977426 10.1210/endo.138.1.4861

[bib26] Lee DH, Esworthy RS, Chu C, Pfeifer GP, Chu FF (2006) Mutation accumulation in the intestine and colon of mice deficient in two intracellular glutathione peroxidases. Cancer Res 66: 9845–9851 17047045 10.1158/0008-5472.CAN-06-0732

[bib27] Lima J, Trovisco V, Soares P, Maximo V, Magalhaes J, Salvatore G, Santoro M, Bogdanova T, Tronko M, Abrosimov A, Jeremiah S, Thomas G, Williams D, Sobrinho-Simoes M (2004) BRAF mutations are not a major event in post-Chernobyl childhood thyroid carcinomas. J Clin Endocrinol Metab 89: 4267–4271 15356020 10.1210/jc.2003-032224

[bib28] Mahoney MC, Lawvere S, Falkner KL, Averkin YI, Ostapenko VA, Michalek AM, Moysich KB, McCarthy PL (2004) Thyroid cancer incidence trends in Belarus: examining the impact of Chernobyl. Int J Epidemiol 33: 1025–1033 15166190 10.1093/ije/dyh201

[bib29] Michiels S, Koscielny S, Hill C (2005) Prediction of cancer outcome with microarrays: a multiple random validation strategy. Lancet 365: 488–492 15705458 10.1016/S0140-6736(05)17866-0

[bib30] Mondello C, Guasconi V, Giulotto E, Nuzzo F (2002) Gamma-ray and hydrogen peroxide induction of gene amplification in hamster cells deficient in DNA double strand break repair. DNA Repair (Amst) 1: 483–493 12509235 10.1016/s1568-7864(02)00035-6

[bib31] Neumann CA, Krause DS, Carman CV, Das S, Dubey DP, Abraham JL, Bronson RT, Fujiwara Y, Orkin SH, Van Etten RA (2003) Essential role for the peroxiredoxin Prdx1 in erythrocyte antioxidant defence and tumour suppression. Nature 424: 561–565 12891360 10.1038/nature01819

[bib32] Nikiforov YE (2002) RET/PTC rearrangement in thyroid tumors. Endocr Pathol 13: 3–16 12114746 10.1385/ep:13:1:03

[bib33] Nikiforov YE, Rowland JM, Bove KE, Monforte-Munoz H, Fagin JA (1997) Distinct pattern of ret oncogene rearrangements in morphological variants of radiation-induced and sporadic thyroid papillary carcinomas in children. Cancer Res 57: 1690–1694 9135009

[bib34] Nikiforova MN, Ciampi R, Salvatore G, Santoro M, Gandhi M, Knauf JA, Thomas GA, Jeremiah S, Bogdanova TI, Tronko MD, Fagin JA, Nikiforov YE (2004) Low prevalence of BRAF mutations in radiation-induced thyroid tumors in contrast to sporadic papillary carcinomas. Cancer Lett 209: 1–6 15145515 10.1016/j.canlet.2003.12.004

[bib35] Ntzani EE, Ioannidis JP (2003) Predictive ability of DNA microarrays for cancer outcomes and correlates: an empirical assessment. Lancet 362: 1439–1444 14602436 10.1016/S0140-6736(03)14686-7

[bib36] Powell N, Jeremiah S, Morishita M, Dudley E, Bethel J, Bogdanova T, Tronko M, Thomas G (2005) Frequency of BRAF T1796A mutation in papillary thyroid carcinoma relates to age of patient at diagnosis and not to radiation exposure. J Pathol 205: 558–564 15714593 10.1002/path.1736

[bib37] Quinn MT, Ammons MC, Deleo FR (2006) The expanding role of NADPH oxidases in health and disease: no longer just agents of death and destruction. Clin Sci (Lond) 111: 1–20 16764554 10.1042/CS20060059

[bib38] Rogounovitch TI, Saenko VA, Ashizawa K, Sedliarou IA, Namba H, Abrosimov AY, Lushnikov EF, Roumiantsev PO, Konova MV, Petoukhova NS, Tchebotareva IV, Ivanov VK, Chekin SY, Bogdanova TI, Tronko MD, Tsyb AF, Thomas GA, Yamashita S (2006) TP53 codon 72 polymorphism in radiation-associated human papillary thyroid cancer. Oncol Rep 15: 949–956 16525684

[bib39] Rosenbaum E, Hosler G, Zahurak M, Cohen Y, Sidransky D, Westra WH (2005) Mutational activation of BRAF is not a major event in sporadic childhood papillary thyroid carcinoma. Mod Pathol 18: 898–902 15968271 10.1038/modpathol.3800252

[bib40] Ruschhaupt M, Huber W, Poustka A, Mansmann U (2004) A compendium to ensure computational reproducibility in high-dimensional classification tasks. Stat Appl Genet Mol Biol 3, article 37 www.bepress.com/sagmb/vol3/iss1/art37/10.2202/1544-6115.1078 16646817

[bib41] Simon R, Radmacher MD, Dobbin K, McShane LM (2003) Pitfalls in the use of DNA microarray data for diagnostic and prognostic classification. J Natl Cancer Inst 95: 14–18 12509396 10.1093/jnci/95.1.14

[bib42] Soares P, Trovisco V, Rocha AS, Lima J, Castro P, Preto A, Maximo V, Botelho T, Seruca R, Sobrinho-Simoes M (2003) BRAF mutations and RET/PTC rearrangements are alternative events in the etiopathogenesis of PTC. Oncogene 22: 4578–4580 12881714 10.1038/sj.onc.1206706

[bib43] Sturgis EM, Zhao C, Zheng R, Wei Q (2005) Radiation response genotype and risk of differentiated thyroid cancer: a case–control analysis. Laryngoscope 115: 938–945 15933498 10.1097/01.MLG.0000163765.88158.86

[bib44] Su AI, Wiltshire T, Batalov S, Lapp H, Ching KA, Block D, Zhang J, Soden R, Hayakawa M, Kreiman G, Cooke MP, Walker JR, Hogenesch JB (2004) A gene atlas of the mouse and human protein-encoding transcriptomes. Proc Natl Acad Sci USA 101: 6062–6067 15075390 10.1073/pnas.0400782101 PMC395923

[bib45] Thomas GA, Williams ED, Becker DV, Bogdanova TI, Demidchik EP, Lushnikov E, Nagataki S, Ostapenko V, Pinchera A, Souchkevitch G, Tronko MD, Tsyb AF, Tuttle M, Yamashita S (2000) Chernobyl tumor bank. Thyroid 10: 1126–1127 11201862 10.1089/thy.2000.10.1126a

[bib46] Tibshirani R, Hastie T, Narasimhan B, Chu G (2002) Diagnosis of multiple cancer types by shrunken centroids of gene expression. Proc Natl Acad Sci USA 99: 6567–6572 12011421 10.1073/pnas.082099299 PMC124443

[bib47] Turner DR, Dreimanis M, Holt D, Firgaira FA, Morley AA (2003) Mitotic recombination is an important mutational event following oxidative damage. Mutat Res 522: 21–26 12517408 10.1016/s0027-5107(02)00194-x

[bib48] Tusher VG, Tibshirani R, Chu G (2001) Significance analysis of microarrays applied to the ionizing radiation response. Proc Natl Acad Sci USA 98: 5116–5121 11309499 10.1073/pnas.091062498 PMC33173

[bib49] United Nations Scientific Committee of the Effect Of Atomic Radiation (2000) Sources, Effect and Risk of Ionizing Radiations. New York: United Nations

[bib50] Williams ED, Abrosimov A, Bogdanova T, Demidchik EP, Ito M, LiVolsi V, Lushnikov E, Rosai J, Sidorov Y, Tronko MD, Tsyb AF, Vowler SL, Thomas GA (2004) Thyroid carcinoma after Chernobyl latent period, morphology and aggressiveness. Br J Cancer 90: 2219–2224 15150580 10.1038/sj.bjc.6601860 PMC2409486

[bib51] Wood RD, Mitchell M, Lindahl T (2005) Human DNA repair genes. Mutat Res 577: 275–283 15922366 10.1016/j.mrfmmm.2005.03.007

[bib52] Wood RD, Mitchell M, Sgouros J, Lindahl T (2001) Human DNA repair genes. Science 291: 1284–1289 11181991 10.1126/science.1056154

[bib53] Xing M (2005) BRAF mutation in thyroid cancer. Endocr Relat Cancer 12: 245–262 15947100 10.1677/erc.1.0978

[bib54] Xiong P, Zheng R, Wang LE, Bondy ML, Shen H, Borer MM, Wei Q, Sturgis EM (2005) A pilot case–control study of gamma-radiation sensitivity and risk of papillary thyroid cancer. Thyroid 15: 94–99 15753665 10.1089/thy.2005.15.94

[bib55] Zhang H, Yu CY, Singer B (2003) Cell and tumor classification using gene expression data: construction of forests. Proc Natl Acad Sci USA 100: 4168–4172 12642676 10.1073/pnas.0230559100 PMC153066

