# Supplementary material for: Genome-wide gene expression profiling suggests distinct radiation susceptibilities in sporadic and post-Chernobyl papillary thyroid cancers
Source: Br J Cancer. 2007 Aug 21;97(6):818–25. doi: 10.1038/sj.bjc.6603938 (PMC2360382; doi:10.1038/sj.bjc.6603938)
Supplement: Supplementary Information [file 6603938x1.doc]

**Supplementary information s3**

**RNA and microarray data preprocessing**

**Tissue processing and isolation of RNA.** Total RNA was extracted using a Trizol reagent kit (Invitrogen, Merelbeke, Belgium) according to the manufacturer’s protocol. The RNA was purified on RNeasy columns (Qiagen, Hilden, Germany) and its integrity was assessed by denaturing agarose gel electrophoresis and by staining of the 18S and 28S RNA bands for visualization. 5 µg of total RNA from each sample were used for *in vitro* transcription amplification (1), following the protocol published by Puskás *et al*. (2), slightly modified: the incubation time for the transcription step was 5 hours instead of 3. This resulted in approximately 20 µg of antisense RNA (aRNA). This procedure was applied to French and CTB samples in the same lab, by the same operator. The only exception are samples V519 and V608 for which the extraction protocol was applied at the CTB. These samples do not stand out on Figure 2, supporting a very limited influence of lab-specific effects, if any.

**RNA labeling and hybridization.** Microarray analyses were performed using commercial Human 1 cDNA Microarray slides (Agilent Technologies, Massy, France). First-strand cDNA was synthesized from 4 µg of aRNA with random primers and amino-allyl-dUTP (Sigma-Aldrich, Bornem, Belgium). Fluorescent dyes (Cy3 and Cy5) coupled with N-hydroxyl-succinimidyl-ester (Amersham Biosciences, Roosendaal, The Netherlands) were used to label the tumoral and the non-tumoral samples, respectively. All hybridizations were replicated with dyes swapped to control for labeling bieses. After stopping the labeling reaction with hydroxylamine 4M (Sigma-Aldrich, Bornem, Belgium), resin column purification with Microcon YM-30 (Millipore, Brussels, Belgium) and ethanol precipitation were realized. The samples were resuspended in 12.5 µl of water. All the tumor/non-tumoral tissue pairs (n=26) were hybridized on Agilent microarray slides according to the manufacturer’s protocol.

**Pre-processing of microarray data**. Microarrays were scanned with a Genepix 4000B scanner. Three scans at decreasing PMT gains were performed for each slide in order to reduce saturation and salvage low intensity spots (3;4). The slide images were quantified with Genepix Pro version 5.0. The resulting fluorescence intensities were processed with functions from the R package for statistics, version 2.1 (5) and from the Bioconductor library version 1.6 (6). Spots below background, or flagged as “bad” by Genepix Pro, for which spot fluorescence cannot be distinguished from the background, were ignored in all pre-processing and analysis steps. After background subtraction, scans at multiple gains were merged with a custom procedure conceptually akin to the method of ref. (3), resulting in an extended expression range. Expression ratios (tumor/normal) were then converted into base-two logarithms. Intensity-dependent and space-dependent biases were removed with the LOESS-based procedures of the marray package for R (7). Color-flip replicates were then averaged. Genes with expression measurement missing for >25% of the samples were not included in the analysis. If the number of missing measurements was <25%, missing data were replaced by 0 (i.e., no differential expression, a conservative assumption). Data are publicly available in the Gene Expression Omnibus database ([www.ncbi.nlm.nih.gov/geo](http://www.ncbi.nlm.nih.gov/geo)), accession number GSE3950.

**Detection of RET/PTC rearrangement.** Reverse transcription was performed on 1µg of total RNA in a final volume of 20µl. Two microliters of the reaction mixture were used for PCR amplification in a final volume of 50µl. The primers 5’-AGGAGATGTACCGCCTGATG-3’ and 5’-GGGGGCATTATTACAGTCCA-3’ (TK) and 5’-CTGCCTGTGCAGTTCTTGTG-3’ and 5’-CCTCGTCGTACACGGTCAC-3’ (EC) were selected by the Primer Express software (Applied Biosystems). Primers for tyrosine kinase domain of RET oncogene (TK) amplified a fragment of 208 bp corresponding to the 5’ part of exon 17, the exon 18 and the 3’ part of exon, 19 spanning a 1075bp and a 1593bp introns. Primers selected for the extracellular domain (EC) amplified a 225bp product corresponding to the 5’ part of exon 3 and the 3’ part of exon 4, spanning a 2323bp intron. PCR reactions consisted in 30 cycles of amplification with the following conditions: 94°C for 45 s, 55°C for 30s, 72°C for 1min. 15µl of PCR products were run on a 2% agarose gel and a direct sequencing was subsequently performed to check the sequence of the PCR products. All the tumors and their adjacent tissues were tested. We used theRNA from the SHSY neuroblastoma cell line as a positive control for both EC and TK PCR reactions and the RNA from theBCPAP cell line as negative control. Samples were considered as positive for a RET/PTC rearrangement when they presented expression of RET TK and no expression of RET EC.

**Detection of V600E (T1799A) BRAF mutation.** BRAF mutation was detected by direct sequencing of exon 15 of BRAF cDNA. Reverse transcription was performed as for RET/PTC rearrangement. PCR primers were designed by the Primer Express software (Applied Biosystems) in exon 15 as follows: 5’-GCACAGGGCATGGATTACTT-3’ and 5’-GATGACTTCTGGTGCCATCC-3’. PCR reactions consisted in 40 cycles of amplification with the following conditions: 94°C for 45s, 55°C for 30s, 72°C for 1 min. The PCR products (195bp) were run on a 2% agarose gel. PCR products were purified with the Qiaquick PCR purification kit (Qiagen, Hilden, Germany) and sequenced in both directions using the primers described earlier. BCPAP cell line was used as a positive control (heterozygous mutation) and the TPC-1 cell line as negative control.

Reference List

1. Van Gelder RN, von Zastrow ME, Yool A, Dement WC, Barchas JD, Eberwine JH. Amplified RNA synthesized from limited quantities of heterogeneous cDNA. Proc Natl Acad Sci U S A 1990; 87(5):1663-1667.

2. Puskas LG, Zvara A, Hackler L, Jr., van Hummelen P. RNA amplification results in reproducible microarray data with slight ratio bias. Biotechniques 2002; 32(6):1330-4, 1336, 1338, 1340.

3. Dudley AM, Aach J, Steffen MA, Church GM. Measuring absolute expression with microarrays with a calibrated reference sample and an extended signal intensity range. Proc Natl Acad Sci U S A 2002; 99(11):7554-7559.

4. Venet D. MatArray: a Matlab toolbox for microarray data. Bioinformatics 2003; 19(5):659-660.

5. R Development Core Team. R: A Language and Environment for Statistical Computing. Vienna, Austria: R Foundation for Statistical Computing, 2004.

6. Gentleman RC, Carey VJ, Bates DM et al. Bioconductor: open software development for computational biology and bioinformatics. Genome Biol 2004; 5(10):R80.

7. Yang YH, Dudoit S, Luu P et al. Normalization for cDNA microarray data: a robust composite method addressing single and multiple slide systematic variation. Nucleic Acids Res 2002; 30(4):e15.
